# Different Levels in Alcohol and Tobacco Consumption in Head and Neck Cancer Patients from 1957 to 2013

**DOI:** 10.1371/journal.pone.0124045

**Published:** 2015-04-13

**Authors:** Yu Zhang, Ruixia Wang, Limin Miao, Longbiao Zhu, Hongbing Jiang, Hua Yuan

**Affiliations:** 1 Jiangsu Key Laboratory of Oral Diseases, Nanjing Medical University, Nanjing, China; 2 Department of Oral and Maxillofacial Surgery, Stomatological Hospital of Jiangsu Province, Nanjing, China; 3 Section of Clinical Epidemiology, Jiangsu Key Lab of Cancer Biomarkers, Prevention and Treatment, Cancer Centre, Nanjing Medical University, Nanjing, China; Duke Cancer Institute, UNITED STATES

## Abstract

**Objective:**

To provide a precise quantification of the association between alcohol and tobacco consumption trends in head and neck cancer patients over the past 45 years.

**Methods:**

We combined findings from all studies published until March 2014 and evaluated the association between different levels in alcohol and tobacco consumption and head and neck cancers through a meta-analytic approach.

**Results:**

We included 28 studies involving 13830 patients with head and neck cancer. In patients with alcohol consumption, the pooled odds ratio (OR) and 95% confidence interval (CI) were 1.29(1.06-1.57), 2.67(2.05-3.48) and 6.63(5.02-8.74) for light drinkers, moderate drinkers and heavy drinkers, respectively. In patients with tobacco consumption, the pooled OR and 95% CI were 2.33(1.84-2.95), 4.97(3.67-6.71) and 6.77(4.81-9.53) for light smokers, moderate smokers and heavy smokers, respectively.

**Conclusion:**

The increased alcohol and tobacco consumption trends increased the risk of head and neck cancer over the past 45 years. Tobacco consumption was found to be a stronger risk factor for head and neck cancer than alcohol consumption. Thus, the control should be considered to limit the intake of alcohol and tobacco.

## Introduction

The broad ranges of alcohol and tobacco consumption patterns around the world create significant public health and safety problems. Convincing evidence demonstrated that alcohol and tobacco consumption increases the risk of cancer in the breast, colorectum, liver, esophagus and head and neck [1–3]. Cigarette contains amounts of chemicals, including many known carcinogens. The carcinogenic role of cigarette varies depending upon the cigarette product. [4]. And the chemical products of alcohol metabolism are also explored to be toxic and hypothesized to cause DNA modifications that lead to cancers [5].

Head and neck cancers (HNC) including cancers in the oral cavity, pharynx and larynx, is one of the most common cancer in the world. There are approximately 300,000 oral cancers, 142,000 pharynx cancers and 157,000 larynx cancers been diagnosed each year [6]. The theory of multiple factors has been well-investigated. Ecological and individual-based studies have reported higher incidence of HNC in deprived populations [7]; lower education and nonprofessional occupations also exhibit a poorer prognosis of HNC [8]; in addition, dietary and nutritional habits have been reported to have a relevant role in the development of HNC [9]. Most of all, amounts of specific research have reported that alcohol and tobacco consumption are the major lifestyle-related risk factors [2,3,10–12]. However, a great amount of the studies with HNC cancers only focused on the intake of alcohol and tobacco, paying little attention on a precise evidence of an association between different levels of alcohol and tobacco consumption. So it is necessary to explore whether different levels of alcohol and tobacco consumption differ the HNC cancer risk. Therefore, we carried out a meta-analysis of epidemiological published studies to quantify the association between different levels of alcohol and tobacco consumption and patients with head and neck cancer.

## Materials and Methods

### Literature search and study inclusion

We carried out a systematic literature search in PubMed and ISI Web of Science for articles published before March 2014. We also reviewed references from reviews, meta-analyses and relevant studies for the sake of completeness. The key words used for the literature search was as follows: (alcohol OR alcoholic beverages) AND (smoke OR tobacco consumption) AND (lip cancer OR tongue cancer OR salivary gland cancer OR gingival cancer OR mouth cancer OR pharynx cancer OR larynx cancer). All titles and abstracts were reviewed by two of the authors.

The articles which met the following explicit criteria were included: 1. Case–control or cohort studies; 2. The exposure of interest were alcohol and tobacco consumption; 3. Risk estimates [odds ratio (OR) or risk ratio (RR)] and corresponding 95% confidence intervals (CIs) or sufficient data to calculate them were reported; 4. Never and/or occasional (non/occasional) drinking was taken as the reference category; 5. At least three levels of alcohol and tobacco consumption and reporting the OR or RR and 95% CIs or sufficient information to calculate them for each level.

### Data extraction

For each included study, we extracted details on publication year, country, study name, cancer site, study design, variables adjusted or matched, risk estimates and 95% CIs. Two reviewers assessed the quality of included articles, and resolved doubts or disagreements. When same studies were published in more than one paper, only the most recent article was included in the analysis.

Since different studies used different units to measure alcohol consumption, we converted it into a uniform measurement (grams per day (g/day)) and the equivalencies was formulate as 0.8 g/ml = 28g/ounce = 12.5 g/drink. The risk estimates of alcohol consumption were separated into light, moderate, and heavy drinkers, which were ≤12.5 g/day, 12.6–49.9 g/day, and ≥50 g/day of alcohol consumption, respectively [13]. Due to the same units of tobacco measurement found in all included studies, we separated tobacco consumption into light, moderate, and heavy smokers, which were ≤19 cigarettes/day, 20–39 cigarettes/day, and ≥40 cigarettes/day of tobacco consumption, respectively. We combined them into a single estimate using the method for pooling non-independent estimates, if more than one value fell into one of these three levels in the study [14].

### Statistical analysis

Because all included publications were case-control studies, we used ORs to measure the interest. When available, we used adjusted risk estimates; otherwise the unadjusted risk estimates were used. Chi-square statistics was calculated to evaluate the heterogeneity across the studies. When P<0.10, the random effect model was selected, otherwise, the fixed effect model was performed [15]. The Egger regression test and Begg-Mazumdar test were used to measure the potential publication bias [16]. We carried out meta-regression models to explored potential sources of between-study heterogeneity [17]. All statistical analyses were carried out in STATA version 12.0. P < 0.05 was considered statistically significant. All *P* values were two-sided.

## Results

### Characteristics of studies

**Fig 1:**
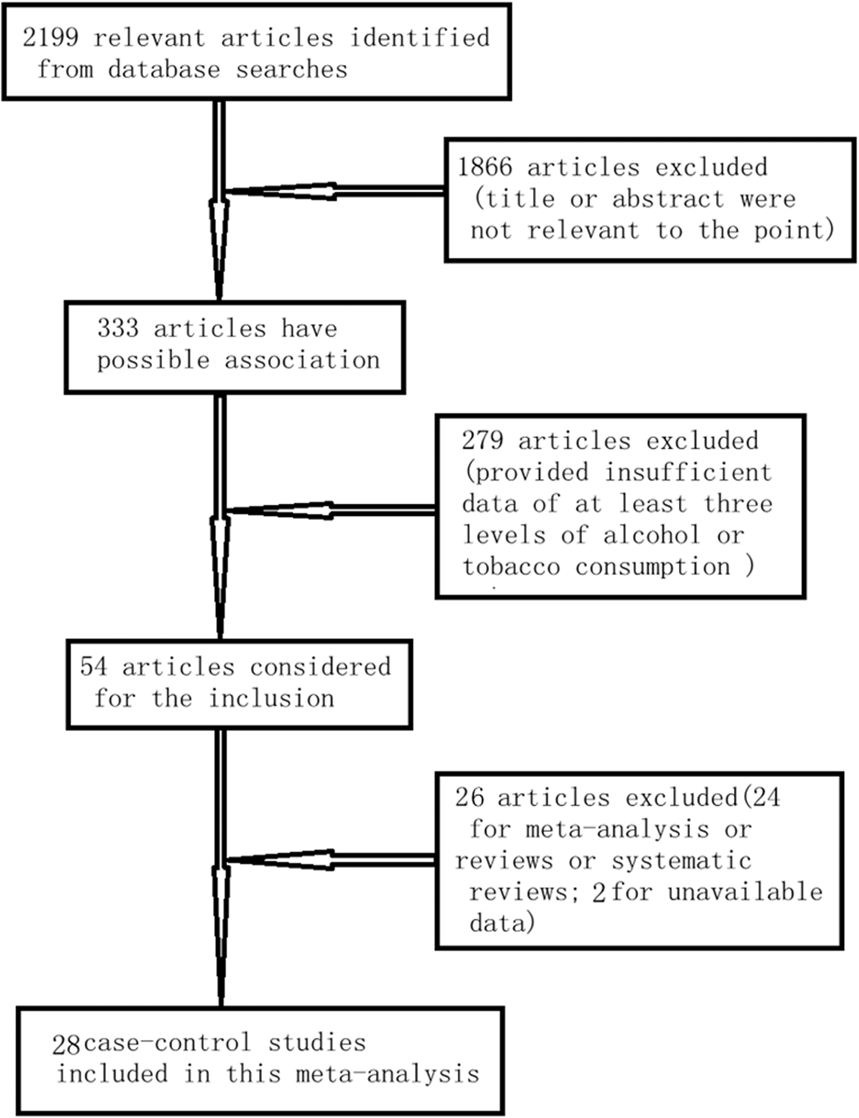
Flowchart of selection of studies for inclusion in meta-analysis.

The process of selection of articles for inclusion was summarized in Fig 1. A total of 2199 relevant articles were identified in initial search, and 28 published studies from 1957 and 2013 were included for this meta-analysis finally. Table 1 showed the main characteristics of the 28 studies [18–45]. A total of 13830 patients with head and neck cancer were included in the meta-analysis. There were 11 studies conducted in America (6 in the USA), 6 in Asia and 11 in Europe.

**Table 1:** Characteristics of 28 studies included in the meta-analysis.

Study	Country	Cases	Sex	Cancer site	Ethnicity	Source of controls	Design
Wynder and Bross [18], 1957	USA	297	MF^a^	Oral, larynx	Mixed	Population based	Case-control
Keller and Terris [19], 1965	USA	544	MF	Oral, larynx	Caucasian	Hospital based	Case-control
Blot et al. [20], 1988	USA	1105	MF	Oral, pharynx	Mixed	Population based	Case-control
Merletti et al. [21], 1989	Italy	122	MF	Oral, pharynx	Caucasian	Population based	Case-control
Zheng et al. [22], 1990	China	165	M ^b^	Oral	Asian	Hospital based	Case-control
Franceschi et al. [23], 1990	Italy	453	MF	Oral, larynx, pharynx	Caucasian	Hospital based	Case-control
Choi and Kahyo [24],1991	Korea	364	MF	Oral, larynx, pharynx	Asian	Hospital based	Case-control
Oreggia et al.[25], 1991	Uruguay	57	MF	Oral	Caucasian	Hospital based	Case-control
Maier et al. [26], 1994	Germany	106	M	pharynx	Caucasian	Population based	Case-control
Hayes et al. [27], 1999	Puerto Rico	321	MF	Oral	Caucasian	Population based	Case-control
Bouchardy et al. [28], 2000	France	257	MF	Oral, larynx, pharynx	Caucasian	Hospital based	Case-control
Garrote et al. [29], 2001	Cuba	160	MF	Oral, pharynx	Mixed	Hospital based	Case-control
Schwartz et al. [30], 2001	USA	326	MF	Oral	Mixed	Population based	Case-control
Zavras et al. [31], 2001	Greece	95	MF	Oral	Caucasian	Hospital based	Case-control
Znaor et al. [32],2003	India	2192	M	Oral, pharynx	Asian	Hospital based	Case-control
Castellsague et al. [33], 2004	Spain	375	MF	Oral, pharynx	Caucasian	Hospital based	Case-control
Menvielle et al. [34], 2004	France	526	MF	Larynx, pharynx	Caucasian	Hospital based	Case-control
Rosenquist et al. [35], 2006	Sweden	128	MF	Oral, pharynx	Caucasian	Population based	Case-control
Vlajinac et al. [36], 2006	Yugoslavia	100	MF	Pharynx	Caucasian	Population based	Case-control
Peters et al. [37], 2006	USA	692	MF	Oral, larynx, pharynx	Mixed	Population based	Case-control
De Stefani et al. [38], 2007	Uruguay	776	MF	Oral, pharynx	Caucasian	Hospital based	Case-control
Applebaum et al. [39], 2007	USA	483	MF	Oral, larynx, pharynx	Mixed	Hospital based	Case-control
Subapriya et al. [40], 2007	India	388	MF	Oral	Asian	Hospital based	Case-control
Oze et al. [41], 2010	Japan	255	MF	Oral, larynx, pharynx	Asian	Hospital based	Case-control
Boing et al. [42], 2011	Brazil	1017	MF	Oral, larynx, pharynx	Caucasian	Hospital based	Case-control
Matsuo et al.[43], 2012	Japan	1009	MF	Oral, larynx, pharynx	Asian	Hospital based	Case-control
Radoı et al. [44], 2012	France	749	MF	Oral	Caucasian	Hospital based	Case-control
Bravi et al.[45], 2013	Italy	768	MF	Oral, pharynx	Caucasian	Hospital based	Case-control

^a^ Male and female

^b^ Male

### Meta-analysis

**Fig 2:**
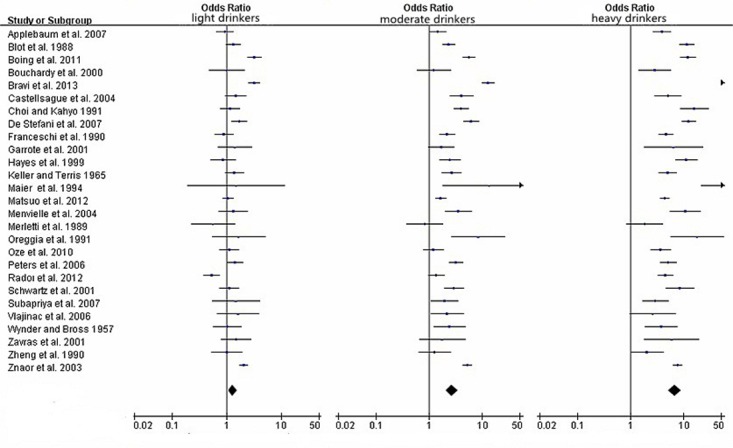
Forest plots for pooled Odds ratios (ORs) and the corresponding 95% confidence intervals (CIs) of HNC cancer risks for light, moderate and heavy drinkers.

**Fig 3:**
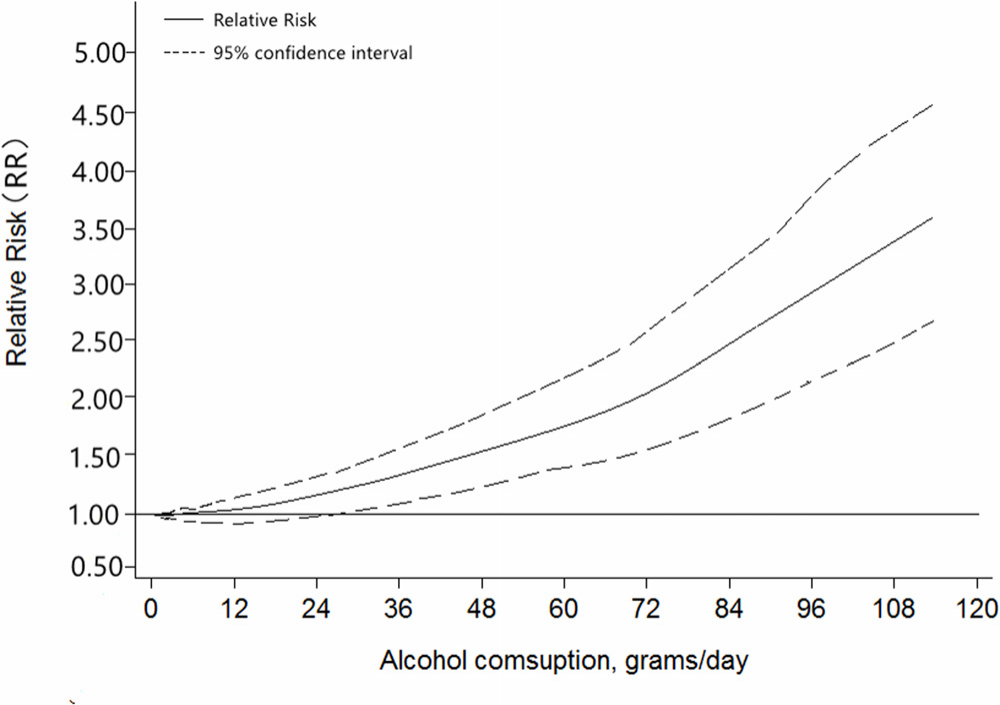
Relative risk function and 95% confidence intervals for the association between alcohol consumption per day and HNC.

Table 2 showed the major results between different levels of alcohol consumption and HNC cancer risk, including the pooled ORs, 95% CIs and heterogeneity. Moreover, in order to obtain a more precise quantification of the association, stratified analyses by cancer type, regional distribution, and gender were performed. Compared with non/occasional drinkers, the pooled ORs and 95%CIs were 1.29(1.06–1.57), 2.67(2.05–3.48) and 6.63(5.02–8.74) for light drinkers, moderate drinkers and heavy drinkers, respectively in Fig 2, with significant heterogeneity between study designs (*I* ^*2*^> 50%). The dose-response analysis was carried out in Fig 3, and the plot also showed the risk of HNC increased with increasing alcohol consumption. As the gender subgroup analysis showed, there were not significant differences in gender in three different levels. However, patients with pharynx cancers seem more susceptive when alcohol consumption increased. At the same time, no susceptibility of cancer risk was found in larynx cancer patients who drink a little. By Geographic area in classification analysis, the association between alcohol drinking and cancer risk was stronger among people from America and Europe than those from Asia.

**Table 2:** Odds ratio (95% confidence interval) of pooled and subgroup analysis for head and neck cancers by alcohol intake.

	Light vs. non/occasional			Moderate vs. non/occasional			Heavy vs. non/occasional		
	OR (95% CI)	*P*-value	*I* ^*2*^	OR (95%CI)	*P*-value	*I* ^*2*^	OR (95%CI)	*P*-value	*I* ^*2*^
**All studies**	1.29(1.06–1.57)	0.010	83%	2.67(2.05–3.48)	<0.001	92%	6.63(5.02–8.74)	<0.001	91%
**Cancer site**									
Oral	1.30(1.14–1.49)	<0.001	0%	2.28(1.68–3.10)	<0.001	87%	3.93(2.78–5.57)	<0.001	93%
pharynx	1.39(1.02–1.89)	0.035	54%	2.87(1.91–4.30)	<0.001	73%	5.70(3.61–9.02)	<0.001	78%
larynx	0.98(0.75–1.29)	0.880	0%	2.06(1.53–2.79)	<0.001	16%	3.00(1.76–5.11)	<0.001	64%
**Gender**									
Male	1.72(1.22–2.44)	0.002	90%	3.00(2.29–3.91)	<0.001	0%	7.46(5.32–10.46)	<0.001	0%
Female	1.60(1.04–2.46)	<0.001	0%	5.37(2.22–13.00)	<0.001	0%	7.84(2.32–26.52)	<0.001	0%
**Geographic area**									
America	1.38(1.06–1.78)	0.020	76%	2.98(2.19–4.06)	<0.001	86%	7.65(5.60–10.45)	<0.001	81%
Europe	1.19(0.72–1.98)	0.500	89%	2.64(1.35–5.14)	0.004	94%	7.36(3.34–13.24)	<0.001	95%
Asia	1.28(0.91–1.82)	0.160	82%	2.22(1.27–3.89)	0.005	95%	4.83(3.15–7.43)	<0.001	89%

**Fig 4:**
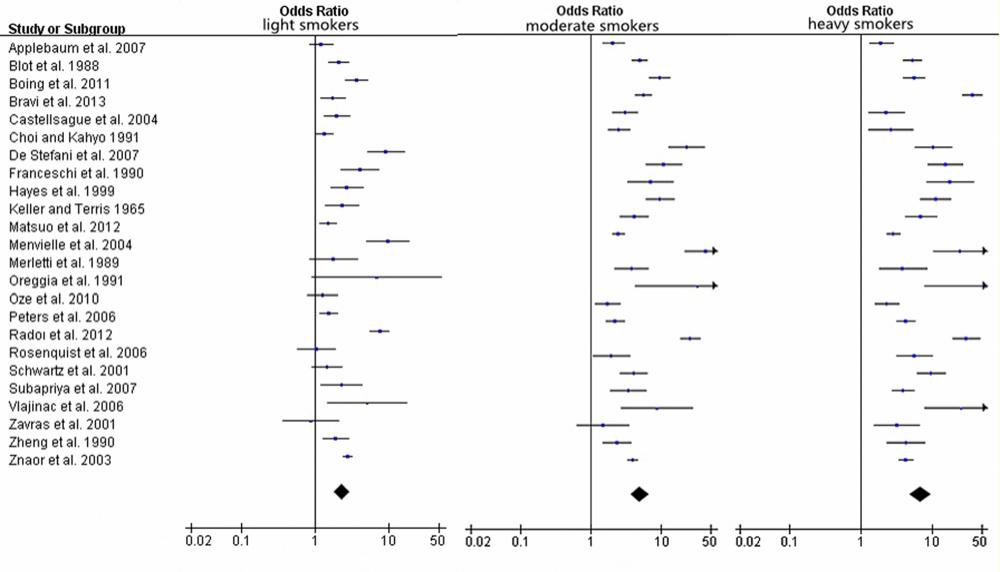
Forest plots for pooled Odds ratios (ORs) and the corresponding 95% confidence intervals (CIs) of HNC cancer risks for light, moderate and heavy smokers.

**Fig 5:**
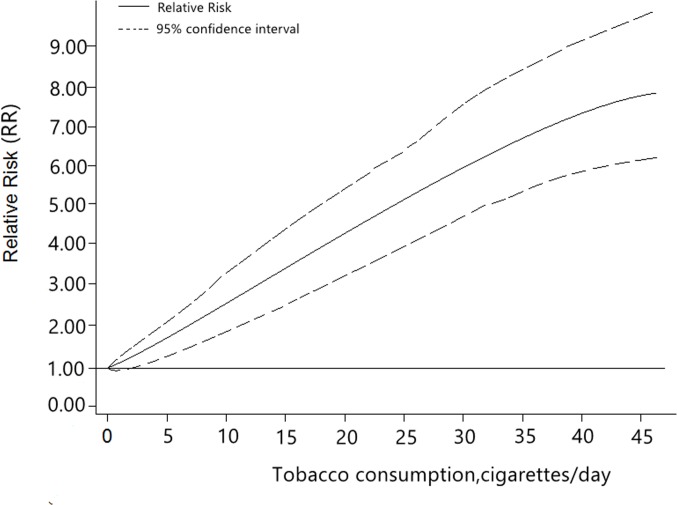
Relative risk function and the 95% confidence intervals for the association between tobacco consumption per day and HNC.

Table 3 showed the characteristics of results between different levels of tobacco consumption and HNC cancer risk. Compared with non/occasional smokers, the pooled ORs and 95%CIs were 2.33(1.84–2.95), 4.97(3.67–6.71) and 6.77(4.81–9.53) for light smokers, moderate smokers and heavy smokers in Fig 4, respectively, with significant heterogeneity between study designs (*I* ^*2*^> 50%). We also carried out a dose-response analysis in Fig 5. The result of the plot showed a significantly increased risk of HNC with increasing tobacco consumption. In female patients, no differences were found in light and moderate levels of tobacco intake, but a higher cancer risk was found with heavy tobacco consumption. By cancer type, larynx cancers seem more susceptive when tobacco intake increased. Moreover, patients of HNC in Europe countries were more susceptive in all three different levels. Tobacco consumption was found to be a stronger risk factor for head and neck cancer than alcohol consumption.

**Table 3:** Odds ratio (95% confidence interval) of pooled and subgroup analysis for head and neck cancers by tobacco intake.

	Light vs. non/occasional			Moderate vs. non/occasional			Heavy vs. non/occasional		
	OR (95% CI)	*P*-value	*I* ^*2*^	OR (95%CI)	*P*-value	*I* ^*2*^	OR (95%CI)	*P*-value	*I* ^*2*^
**All studies**	2.33(1.84–2.95)	<0.001	88%	4.97(3.67–6.71)	<0.001	94%	6.77(4.81–9.53)	<0.001	94%
**Cancer site**									
Oral	1.51(1.19–1.93)	0.001	84%	2.79(1.93–4.04)	<0.001	91%	4.00(2.57–6.22)	<0.001	92%
pharynx	2.07(1.28–3.34)	0.003	74%	3.52(2.03–6.11)	<0.001	78%	5.72(3.34–9.80)	<0.001	73%
larynx	2.89(1.81–4.61)	<0.001	0%	5.37(3.33–8.67)	<0.001	0%	11.30(6.26–20.39)	<0.001	0%
**Gender**									
Male	1.68(1.19–2.38)	<0.001	32%	3.25(1.94–5.44)	<0.001	88%	4.03(2.45–6.62)	<0.001	41%
Female	1.62(0.69–3.78)	0.267	62%	3.66(2.45–5.48)	<0.001	0%	7.80(3.04–19.96)	<0.001	17%
**Geographic area**									
America	2.44(1.66–3.58)	<0.001	85%	5.78(3.50–9.53)	<0.001	92%	6.99(4.72–10.33)	<0.001	86%
Europe	2.74(1.53–4.92)	<0.001	91%	7.20(3.37–15.37)	<0.001	94%	10.48(4.89–22.49)	<0.001	94%
Asia	1.78(1.27–2.50)	<0.001	87%	2.67(2.03–3.52)	<0.001	80%	3.29(2.64–4.10)	<0.001	58%

For the head and neck cancer is a chronic process, the duration of alcohol and tobacco consumption should be considered for a risk factor. So we showed results of pooled and subgroup analysis for head and neck cancers by years of cigarette smoking. As shown in Table 4, the pooled ORs and 95%CIs were 1.93(1.37–2.73), 3.39(2.47–4.66) and 3.39(2.47–4.66) for patients who smoke for 1–19 years, 20–40 years and>40 years, respectively. Unfortunately, we could not get enough data of the duration of alcohol consumption.

**Table 4:** Odds ratio (95% confidence interval) of pooled and subgroup analysis for head and neck cancers by years of cigarette smoking.

	1–19 years			20–39 years			>40 years		
	OR (95% CI)	*P*-value	*I* ^*2*^	OR (95%CI)	*P*-value	*I* ^*2*^	OR (95%CI)	*P*-value	*I* ^*2*^
**All studies**	1.93(1.37–2.73)	<0.001	82%	3.39(2.47–4.66)	<0.001	78%	5.17(3.93–6.80)	<0.001	70%
**Cancer site**									
Oral	1.77(1.21–2.61)	0.004	46%	3.33(2.07–5.37)	<0.001	68%	4.73(1.70–8.29)	<0.001	76%
pharynx	2.09(1.08–4.05)	0.029	69%	3.29(1.18–9.13)	0.022	86%	6.03(2.34–15.54)	<0.001	83%
larynx	3.18(1.70–5.97)	<0.001	0%	4.13(2.25–7.58)	<0.001	0%	9.32(4.06–21.42)	<0.001	50%
**Gender**									
Male	1.57(0.86–2.56)	0.138	75%	3.34(1.73–6.43)	<0.001	77%	4.94(2.84–8.61)	<0.001	69%
Female	1.79(0.99–3.27)	0.056	46%	3.26(2.21–4.80)	<0.001	0%	4.35(2.88–6.59)	<0.001	0%
**Geographic area**									
America	1.75(0.99–3.11)	0.055	91%	3.35(2.10–5.34)	<0.001	86%	5.54(3.96–7.74)	<0.001	73%
Europe	2.86(1.81–4.52)	<0.001	27%	4.91(2.56–9.41)	<0.001	64%	7.46(4.18–13.29)	<0.001	52%
Asia	1.52(1.12–2.06)	0.008	0%	2.14(1.53–3.01)	<0.001	0%	2.80(2.00–3.92)	<0.001	0%

### Sensitivity analysis and publication bias

In order to compare the difference and evaluate the sensitivity of the meta-analyses, we repeated the meta- analysis with one study excluded at each time. The results did not have significant change of the pooled OR even if the most influential study was omitted. We also excluded studies by different subgroup (cancer site, geographic area, sample size and study time), and we did not find any significant change of the pooled OR (data not shown). These indicated the robustness of our findings. Funnel plot and Egger’s test were performed to assess the publication bias. The shape of the funnel plot (S1–S6 Figs) did not show any obvious evidence of asymmetry for *P* = 0.070, *P* = 0.154, *P* = 0.960, *P* = 0.899, *P* = 0.274, and *P* = 0.139, respectively.

## Discussion

The results of the present meta-analysis, based on 13,830 HNC cases, provide quantitative testimony for a positive association between alcohol and tobacco consumption and HNC risks. Compared with non/occasional drinkers, we found that alcohol consumption was associated with a significantly increased risk at moderate and heavy levels for HNC patients, while our results showed much higher ORs of pharynx cancer compared with those presented among patients with oral and larynx cancers. Moreover, our results were rather similar to those shown in previous studies [46, 47]. The association between alcohol consumption and cancer risk was stronger among people from America and Europe than those from Asia. One possible theory was that the proportion of moderate and heavy drinkers was higher in America and Europe population. Another possible theory was that people from America and Europe carried a higher prevalence of the polymorphisms of alcohol metabolism related genes, such as aldehyde dehydrogenase (ALDH) and alcohol dehydrogenase (ADH), having a lower risk of some alcohol-related cancer consequently [48,49].

A significantly increased risk at all three different levels was found from the results of the tobacco consumption meta-analysis. It showed a significantly higher increased risk for larynx cancers than oral and pharynx cancer. One of the possible theory was that fibers of tobacco could be released and then penetrated into the laryngeal tissue, thus becoming a further risk factor initiating tumorigenesis [50]. Another possible reason was that the number of studies from larynx is limited. Meanwhile, patients with tobacco smoking habit in Europe countries seemed more susceptive with HNC risks.

In some epidemiologic studies, ethanol had been described as a risk enhancer in smokers, but not as an independent risk factor [51]. However, most of the studies provided strong evidence that alcohol consumption, independently from exposure to tobacco consumption, increased the risk of head and neck cancer [20,46,52,53]. Unfortunately, we did not get enough data between alcohol and tobacco consumption in patients with HNC, the analysis of the alcohol and tobacco interaction was not explored.

Limitations of our meta-analysis include some possible residual confounding, such as diet, physical activity and human papillomavirus (HPV) infection. But we could not ignore and exclude these residual confounding. Some studies demonstrated that HPV infection is a risk factor for HNC cancer, especially for cancers of the tongue [54,55]. However, from the result of other different studies, whether the associations between alcohol and tobacco consumption and HNC cancer risk differ according to HPV status was not clear [39,56,57]. Nevertheless, significant heterogeneity among 28 included studies was observed, which possibly due to the study design and quality. However, random-effects model was allowed to be used into compute the heterogeneity [17]. Additionally, the lifetime of patients’ exposure to alcohol or tobacco and the different type of alcoholic and smoking beverage were not studied in this meta-analysis. Recall bias was another possible limitation of our study, we paid attention to collect the value of alcohol and tobacco consumption in a standardized manner, but differential reporting between articles could not be excluded.

In conclusion, tobacco consumption was found to be a stronger risk factor for head and neck cancer than alcohol consumption. The pharynx was the most affected by the harmful effects of alcohol, while larynx was the most affected by the harmful effects of tobacco. Europeans should pay more attention to harmful effects of tobacco. Tobacco consumption increased the risk of head and neck cancer even for smaller quantities whereas alcohol drinking increased this risk significantly at moderate and heavy levels. Precancerous lesions or cancer at an early stage should be detected by regular check-ups. Prevention efforts should be focused on smoking and drinking cessation [58].

## Supporting Information

S1 Fig Funnel plot of publication bias in light drinkers vs. non/occasional. (TIF)

S2 Fig Funnel plot of publication bias in moderate drinkers vs. non/occasional. (TIF)

S3 Fig Funnel plot of publication bias in heavy drinkers vs. non/occasional. (TIF)

S4 Fig Funnel plot of publication bias in light smokers vs. non/occasional. (TIF)

S5 Fig Funnel plot of publication bias in moderate smokers vs. non/occasional. (TIF)

S6 Fig Funnel plot of publication bias in heavy smokers vs. non/occasional. (TIF)

S1 Table Summary statistics for the association between tobacco intake and HNC risk in strata of selected covariates. (DOCX)

S2 Table Summary statistics for the association between alcohol intake and HNC risk in strata of selected covariates. (DOCX)
