# Supplementary material for: Different Levels in Alcohol and Tobacco Consumption in Head and Neck Cancer Patients from 1957 to 2013
Source: PLoS One. 2015 Apr 13;10(4):e0124045. doi: 10.1371/journal.pone.0124045 (PMC4395416; doi:10.1371/journal.pone.0124045)
Supplement: S1 Table — (DOCX) [file pone.0124045.s007.docx]

S1 Table. Summary statistics for the association between tobacco intake and HNC risk in strata of selected covariates.

|  | Light vs. non/occasional | | | Moderate vs. non/occasional | | | Heavy vs. non/occasional | | |
| --- | --- | --- | --- | --- | --- | --- | --- | --- | --- |
|  | OR (95% CI) | *P*-value | *I^2^* | OR (95% CI) | *P*-value | *I^2^* | OR (95% CI) | *P*-value | *I^2^* |
| Cancer site |  |  |  |  |  |  |  |  |  |
| Oral | 1.51(1.19-1.93) | 0.001 | 84% | 2.79(1.93-4.04) | <0.001 | 91% | 4.00(2.57-6.22) | <0.001 | 92% |
| Pharynx | 2.07(1.28-3.34) | 0.003 | 74% | 3.52(2.03-6.11) | <0.001 | 78% | 5.72(3.34-9.80) | <0.001 | 73% |
| Larynx | 2.89(1.81-4.61) | <0.001 | 0% | 5.37(3.33-8.67) | <0.001 | 0% | 11.30(6.26-20.39) | <0.001 | 0% |
| *P* _heterogeneity_ between strata =0.001 | | | | *P* _heterogeneity_ between strata < 0.001 | | | *P* _heterogeneity_ between strata < 0.001 | | |
| Gender |  |  |  |  |  |  |  |  |  |
| Male | 1.68(1.19-2.38) | <0.001 | 32% | 3.25(1.94-5.44) | <0.001 | 88% | 4.03(2.45-6.62) | <0.001 | 41% |
| Female | 1.62(0.69-3.78) | <0.267 | 62% | 3.66(2.45-5.48) | <0.001 | 0% | 7.80(3.04-19.96) | <0.001 | 17% |
| *P* _heterogeneity_ between strata =0.002 | | | | *P* _heterogeneity_ between strata < 0.001 | | | *P* _heterogeneity_ between strata < 0.001 | | |
| Geographic area | |  |  |  |  |  |  |  |  |
| America | 2.44(1.66-3.58) | <0.001 | 85% | 5.78(3.50-9.53) | <0.001 | 92% | 6.99(4.72-10.33) | <0.001 | 86% |
| Europe | 2.74(1.53-4.92) | <0.001 | 91% | 7.20(3.37-15.37) | <0.001 | 94% | 10.48(4.89-22.49) | <0.001 | 94% |
| Asia | 1.78(1.27-2.50) | <0.001 | 87% | 2.67(2.03-3.52) | <0.001 | 80% | 3.29(2.64-4.10) | <0.001 | 58% |
| *P* _heterogeneity_ between strata < 0.001 | | | | *P* _heterogeneity_ between strata < 0.001 | | | *P* _heterogeneity_ between strata < 0.001 | | |
