# Supplementary material for: Different Levels in Alcohol and Tobacco Consumption in Head and Neck Cancer Patients from 1957 to 2013
Source: PLoS One. 2015 Apr 13;10(4):e0124045. doi: 10.1371/journal.pone.0124045 (PMC4395416; doi:10.1371/journal.pone.0124045)
Supplement: S2 Table — (DOCX) [file pone.0124045.s008.docx]

S2 Table. Summary statistics for the association between alcohol intake and HNC risk in strata of selected covariates.

|  | Light vs. non/occasional | | | Moderate vs. non/occasional | | | Heavy vs. non/occasional | | |
| --- | --- | --- | --- | --- | --- | --- | --- | --- | --- |
|  | OR (95% CI) | *P*-value | *I^2^* | OR (95% CI) | *P*-value | *I^2^* | OR (95% CI) | *P*-value | *I^2^* |
| Cancer site |  |  |  |  |  |  |  |  |  |
| Oral | 1.30(1.14-1.49) | <0.001 | 0% | 2.28(1.68-3.10) | <0.001 | 87% | 3.93(2.78-5.57) | <0.001 | 93% |
| Pharynx | 1.39(1.02-1.89) | 0.035 | 54% | 2.87(1.91-4.30) | <0.001 | 73% | 5.70(3.61-9.02) | <0.001 | 78% |
| Larynx | 0.98(0.75-1.29) | 0.880 | 0% | 2.06(1.53-2.79) | <0.001 | 16% | 3.00(1.76-5.11) | <0.001 | 64% |
| *P* _heterogeneity_ between strata=0.032 | | | | *P* _heterogeneity_ between strata < 0.001 | | | *P* _heterogeneity_ between strata < 0.001 | | |
| Gender |  |  |  |  |  |  |  |  |  |
| Male | 1.72(1.22-2.44) | 0.002 | 90% | 3.00(2.29-3.91) | <0.001 | 0% | 7.46(5.32-10.46) | <0.001 | 0% |
| Female | 1.60(1.04-2.46) | <0.001 | 0% | 5.37(2.22-13.00) | <0.001 | 0% | 7.84(2.32-26.52) | <0.001 | 0% |
| *P* _heterogeneity_ between strata < 0.001 | | | | *P* _heterogeneity_ between strata < 0.001 | | | *P* _heterogeneity_ between strata < 0.001 | | |
| Geographic area | |  |  |  |  |  |  |  |  |
| America | 1.38(1.06-1.78) | 0.020 | 76% | 2.98(2.19-4.06) | <0.001 | 86% | 7.65(5.60-10.45) | <0.001 | 81% |
| Europe | 1.19(0.72-1.98) | 0.500 | 89% | 2.64(1.35-5.14) | 0.004 | 94% | 7.36(3.34-13.24) | <0.001 | 95% |
| Asia | 1.28(0.91-1.82) | 0.160 | 82% | 2.22(1.27-3.89) | 0.005 | 95% | 4.83(3.15-7.43) | <0.001 | 89% |
| *P* _heterogeneity_ between strata =0.005 | | | | *P* _heterogeneity_ between strata < 0.001 | | | *P* _heterogeneity_ between strata < 0.001 | | |
